# The correlation between yttria content and thermal–mechanical properties of GRX-810

**DOI:** 10.1007/s40964-026-01834-1

**Published:** 2026-07-10

**Authors:** Yehong Chen, Huan Ding, Selami Emanet, Ehsan Bagheri, Michael M. Talachian, Paul R. Gradl, Shengmin Guo

**Affiliations:** 1 Department of Mechanical and Industrial Engineering, Louisiana State University, Baton Rouge, USA; 2 NASA Marshall Space Flight Center, Huntsville, USA

**Keywords:** L-PBF, GRX810, Thermal-property, HIP, Mechanical-property

## Abstract

In this paper, laser powder bed fusion (L-PBF) prepared GRX-810 samples with different yttria contents (from 0.3 to 1 wt%) were systematically examined. The mechanical properties and thermal properties were measured under as-built and Hot Isostatic Pressing (HIP) heat treated conditions. The results indicate that yttria content significantly affects the thermal properties of as-built L-PBF GRX-810. Higher yttria reduces thermal diffusivity and conductivity by up to 6% in of as-built samples. HIP restores thermal properties to low-yttria levels, indicating that L-PBF-induced microdefects and porosity, rather than composition, primarily cause the thermal degradation. Based on the mechanical testing results, yttria content up to 0.45% provides the most favorable strength–ductility balance in the present dataset. Higher yttria content (from 0.5 to 1%) leads to the reduction of the elongation from 27% to around 4% in the as-built samples. Following HIP, the yield strength decreases from ~ 785 MPa in the as-built state to ~ 685 MPa. The elongation increases from 4% in the as-built state to ~ 42%. HIP samples with yttria content less than 0.45% exhibit a high ductility at approximately 40% maximum elongation, while HIP samples with yttria content higher than 0.5% exhibit a moderate ductility of approximately 18% maximum elongation at failure. These thermal and mechanical performance studies provide valuable guidance for GRX-810 applications and provide a reference for further AM-ODS alloy optimization.

## Introduction

NASA recently developed a new oxide-dispersion-strengthened (ODS) NiCoCr-based alloy, employing a model-driven alloy design methodology and utilizing laser powder bed fusion (L-PBF) based additive manufacturing (AM) technologies. This innovative alloy is designated GRX-810. NiCoCr alloys have attracted significant attention in recent years due to their excellent mechanical properties, thermal stability, and corrosion resistance [1–5]. Ni-based alloys’ stable mechanical strength at high temperatures and superb creep resistance make them the perfect candidate materials for energy generation applications, aerospace applications, heat exchangers, and high temperature industrial processing and petrochemical applications. Current research topics on NiCoCr alloys include the mechanisms of nanoparticle dispersion strengthening, and how grain refinement and compositional adjustments would impact mechanical and thermal performance, etc. Studies on AM of novel NiCoCr alloys are also popular topics, aiming to overcome the limitations of traditional manufacturing methods for fabrication of complex and precisely engineered components [6–13].

GRX-810 alloy is mainly composed of Ni, Co, and Cr and further enhanced by yttria nanoparticles, which was first disclosed in the 2020s [1]. NASA researchers revealed how AM could be leveraged to produce ODS alloy GRX-810 without the use of traditional resource-intensive processing steps such as mechanical alloying or chemical reactions. The GRX-810 alloy was specifically designed for aerospace applications, including liquid rocket engine injectors, preburners, turbines, and hot-section components, capable of withstanding temperatures up to 1,316 °C. Consequentially, GRX-810 represents a class of alloys that can bridge the temperature gap between traditional nickel-based superalloys and refractory alloys. Resonant acoustic mixing was employed to coat NiCoCr based powders with nano-scale yttrium oxides. Then, through the L-PBF AM process, the coated powders were successfully consolidated into 99.9% dense parts. Due to the high temperature and extremely strong mixing in the laser molten tracks, and fast cooling-rate of the L-PBF process (10^6^–10^7^ K/s), nano-scale yttrium oxides can be dispersed into the NiCoCr matrix. Taking the benefit of dispersing yttria nanoparticles into the alloy matrix, GRX-810 alloy is effectively strengthened by yttria for hindering the movement of dislocations and enhancing grain boundary stability at high temperatures. NASA researchers demonstrated that the mechanical results of GRX-810 had a twofold improvement in strength, over 1,000-fold better creep performance, and twofold improvement in oxidation resistance compared with the traditional polycrystalline wrought Ni-based alloys at 1093 °C [1].

Yttria is well known for its high melting point (above 2400 °C) and exceptional stability, which makes it an ideal candidate for high temperature strengthening in metal alloys. The incorporation of yttria nanoparticles uniformly in the base alloy matrix is the crucial factor for improving the performance of GRX-810. Element yttrium is widely known for refining the microstructures and enhancing the extreme temperature stability of alloys [14, 15]. The yttria nanoparticles can inhibit the movement of dislocations which is usually the primary reason for material failure under high stress [16–22]. Creep resistance is significantly crucial to the materials utilized under high temperature and large stress environments, where the conventional materials might deform slowly over time. The integration of yttria nanoparticles can enhance an alloys’ creep resistance. This dispersion strengthening mechanism could significantly extend the service life and durability of GRX-810, particularly in the applications that require long term performance in harsh environments.

Hot Isostatic Pressing (HIP) is a critical thermomechanical process used to optimize the alloy performance. HIP exposes parts simultaneously to high temperatures and uniform gas pressure (often exceeding 100 MPa) in an inert gas environment, a process effectively removing porosities and micro-defects. HIP can improve thermal conductivity, ductility, and structural consistency by enhancing interparticle bonding [23–26]. Although HIP provides these advantages, it may also result in yield strength reduction due to grain coarsening and detrimental phase formation and aging. For example, Kreitcberg et al. [27] systematically investigated the mechanical behavior of L-PBF processed Inconel 625 in the non-heat treated (NHT) state and after different post-processing routes, including HIP. Compared with the NHT, HIP effectively eliminated process-induced micro-porosity and homogenized the microstructure through recrystallization, which also led to grain coarsening and the formation of MC carbides. More importantly, HIP resulted in a pronounced improvement in tensile ductility: at 760 °C, the elongation to failure of horizontally built samples increased from ~ 5 to 7% in the NHT condition to ~ 25% after HIP, while the elongation of vertically built samples was maintained at ~ 22–25%, thereby largely suppressing the build-orientation dependence of tensile properties. Poulin et al. [28]. reported that a HIP treatment of L-PBF Inconel 625 for 4 h at 1120 °C under a 100 MPa argon atmosphere significantly reduced the porosity from (0.06, 0.25, 0.87, and 2.67%) down to (0.03, 0.06, 0.08, and 0.38%). Xu et al. [29] examined the impact of HIP (1200 °C and 150 MPa for 4 h) on mechanical properties of L-PBF Inconel 738 alloy. HIP reduced the defect densities in the XY and YZ planes of the as-built Inconel 738 alloy from (0.53% and 0.292%) to (0.251 and 0.123%). The HIP treatment also reduced the room temperature tensile properties of the as-built Inconel 738 alloy because of grain growth, low dislocation density, and coarser precipitates. However, HIP treatments significantly improved the ductility under room temperature tensile testing.

Currently, the effect of yttria loading in GRX-810 is not well understood. A uniform dispersion of an appropriate amount of yttria in the base alloy matrix is necessary for achieving desirable mechanical performance in GRX-810. However, under the same L-PBF laser melting strategies, different amounts of yttria powders may behave differently with the NiCoCr powders in terms of melting, mixing, and redistribution. As a result, different yttria contents could lead to distinct thermal, electrical, and mechanical properties in NHT L-PBF components. Therefore, a natural research question is how yttria content affects the properties and microdefect formation in L-PBF GRX-810 components.

In this study, GRX-810 alloys with different yttria contents were examined under both L-PBF AM NHT condition and after-HIP condition. Specifically, by varying the yttria content over the range from 0.3 to 1.0 wt%, this experimental study evaluated the mechanical and thermal properties of L-PBF GRX-810 alloys. The yttria composition matrix used in this study was treated as a literature-informed screening matrix within the GRX-810 alloy development framework, rather than a uniformly spaced statistical design-of-experiments matrix. The selected compositions cover both higher yttria contents comparable to previously reported GRX-810/AM-ODS compositions and a more densely sampled low-yttria region (0.30–0.45 wt% Y_2_O_3_), where the present results reveal a more favorable strength–ductility balance. Therefore, the current dataset should be interpreted as a guided exploratory screening of available yttria compositions, rather than as a statistically optimized composition design. More specifically, the objective of this work is to evaluate how yttria content and HIP treatment influence the thermal and mechanical properties of L-PBF GRX-810 alloys and to establish a preliminary processing–microstructure–property correlation based on measured property trends and representative microstructural observations. Representative microstructural analyses were also performed to support the interpretation of the observed property trends, while a fully quantitative microstructural investigation is reserved for a dedicated follow-up study.

## Materials and methods

### Materials and sample preparation

**Fig. 1:**
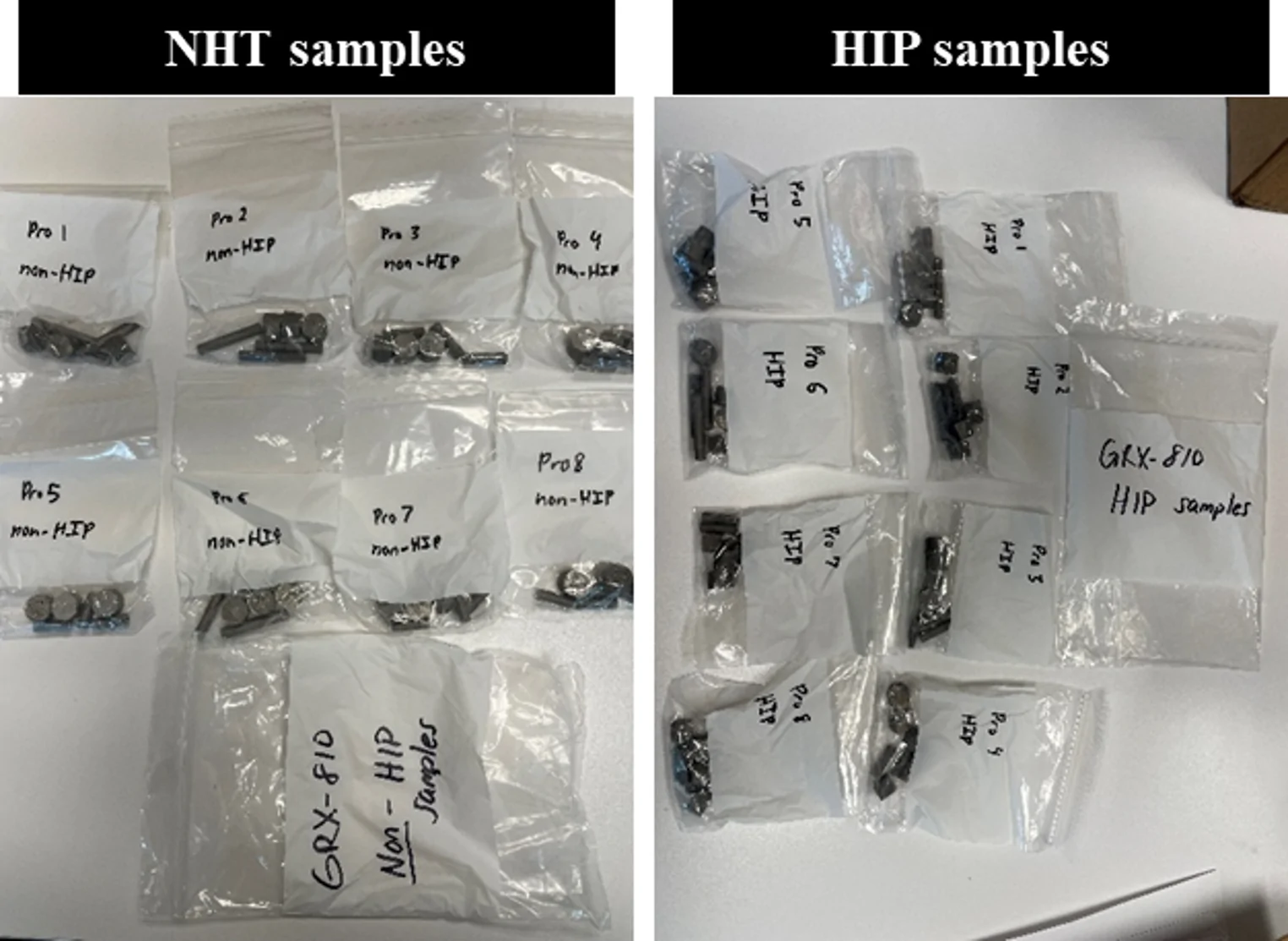
Eight sets of GRX-810 L-PBF samples with different yttria content, NHT and HIP processed

In this study, eight sets of GRX-810 samples with different yttria content were produced by using L-PBF on an EOS M280 machine, Fig. 1. The L-PBF prepared samples were separated under NHT and HIP treated subgroups. For each subgroup, there are three cylindrical shaped samples around 20 mm in length and 6 mm in diameter, which are used for tensile testing, and three 12.7 mm diameter disks for thermal property measurements. The L-PBF process was conducted with a volumetric energy density of 57–86 J/mm^3^. The nominal composition for base metal powder GRX-810 is listed in Table 1. And the yttria addition is listed in Table 2. The GRX-810 samples used in this study were prepared by NASA following the established GRX-810 powder preparation route reported by Smith et al. [1]. In that process, nanoscale Y_2_O_3_ particles were coated onto the NiCoCr-based alloy powder using a high-energy acoustic mixer, without the use of binders, fluids, or chemical reactions. The prior work also showed that this coating process preserved the spherical morphology of the metal powder, and the post-mixed powder was sieved to remove large oxide or metallic particles. Therefore, the yttria contents listed in Table 2 are reported as nominal designed additions before L-PBF processing. Direct quantification of yttria loss or retained yttria fraction after powder handling and laser processing was not performed in the present study; thus, all composition-dependent comparisons are discussed based on nominal yttria content. The yttria contents listed in Table 2 represent the nominal composition matrix used for this screening study. Because this matrix was not designed as a uniform design-of-experiments sequence, the present work focuses on comparing the thermal and mechanical responses of these available compositions and identifying composition ranges that merit more detailed future optimization.

**Table 1:** Nominal composition for base powder GRX-810

Element	Al	C	Cr	Co	Nb	Ni	Re	Ti	W
Nominal (wt%)	0.3	0.055	30	32	0.8	Balance	1.5	0.3	3.0

The HIP samples were processed at 1163 °C for 3–4 h at 104 MPa and these HIP conditions are commonly used for processing standard nickel based superalloys (according to ASTM standard F3301-18a) [30].

**Table 2:** Nominal yttria ratios (wt%) for the tested GRX-810 L-PBF samples before L-PBF

Sample number	1	2	3	4	5	6	7	8
Yttria	1	0.9	0.7	0.5	0.3	0.45	0.4	0.35

### The apparatus and theory for thermal diffusivity/conductivity measurements

**Fig. 2:**
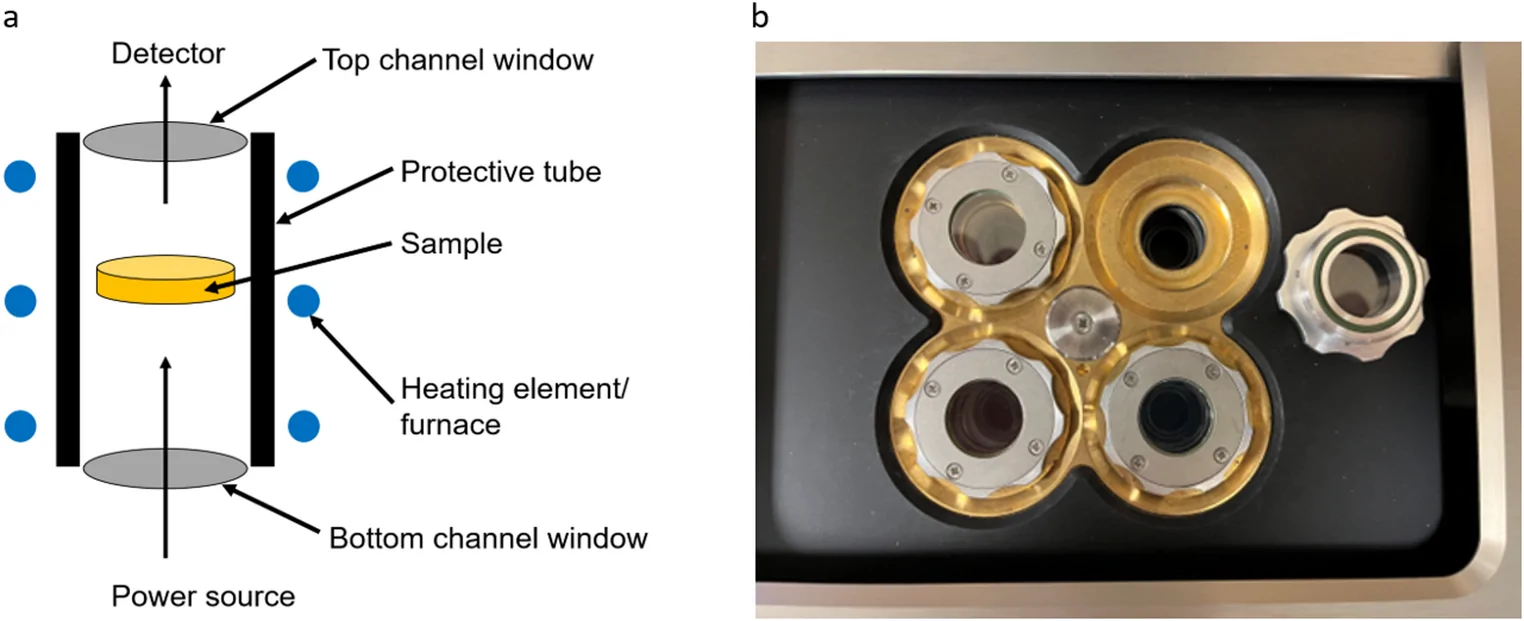
**a** Schematic diagram of Netzsch LFA 467 HT apparatus. **b** LFA 467 HT has four testing cells. The top window of the top-right cell is removed for sample installation

Thermal diffusivity and conductivity measurements were conducted using a Netzsch LFA 467 HT apparatus, Fig. 2. Netzsch LFA 467 HT operates in a vertical orientation, with a pulsed energy (xenon light pulse) introduced through the bottom window toward the sample. The sample is centrally positioned, while an infrared (IR) detector is located above the top window. A xenon lamp serves as the flash source, controlled by software to deliver pulses ranging from 20 to 1200 µs. After the irradiation of the sample’s lower surface by the xenon lamp flash, the IR detector measures the temperature increase on the opposite side as a function of time [31]. The thermal diffusivity of the sample is calculated from the measured temperature-time curve using Eq. 1 [32].

1 $$a=0.1388\:\times\:\frac{{l}^{2}}{{t}_{1/2}}$$

*a* is the thermal diffusivity in mm^2^/s.

*l* is the thickness of the test piece in mm.

*t*_1/2_ is the time (in second, after the flash pulse) at 50% of the temperature increase, measured on the top surface of the test piece.

As shown in Fig. 2b, Netzsch LFA 467 HT can load up to four test samples. The L-PBF GRX-810 samples were first machined (turning and EDM) for thermal property testing. The disk-shaped samples were specified to have a diameter of 12.40 ± 0.05 mm, and a thickness of 1.80 ± 0.05 mm. After machining, the top/bottom surfaces of the disks were ground using SiC papers (320 and 600 grit, successively). Prior to the thermal property tests, the sample density (*ρ*) was measured by weighing the weight of each sample and dimensional measurement using a digital micrometer at room temperature. After cleaning with ethanol, the surfaces of the reference sample (a 304 stainless-steel material from NETZSCH corp.), and each target sample were coated with a thin and uniform layer of dry graphite to ensure identical heat absorption. For each run, typically three samples from a subgroup and one reference sample were loaded into Netzsch LFA 467 HT. Thermal properties of the GRX-810 AM samples were evaluated from room temperature (25 °C) to 900 °C at a heating rate of 25 °C /min, with a dwell time of ~ 60 min at each testing temperature. After obtaining specific heat and density values, thermal conductivity is calculated using the following equation:

2 $$K\left(T\right)=\:{C}_{p}\left(T\right)\times\:a\left(T\right)\times\:\rho\:\left(T\right)$$

### Mechanical tensile testing

**Fig. 3:**
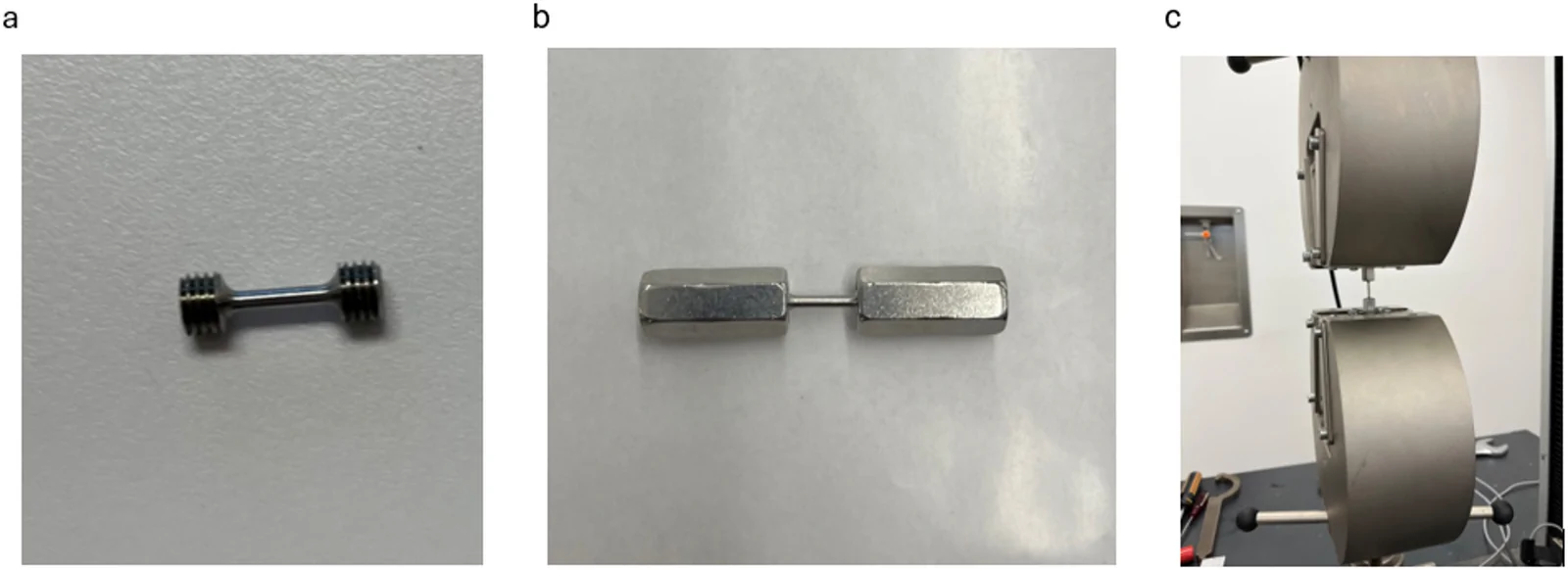
**a** Tensile sample without holder, **b** sample with holders, **c** sample mounted on a tensile test machine

The GRX-810 alloy tensile test samples were prepared from cylindrical shaped L-PBF samples using a lathe. The sample design was referenced to the Standard Test Methods for Tension Testing of Metallic Materials [33]. The gauge section length was 6.35 mm and the two ends of the tensile testing samples were machined into the threaded ends with 1/4-28 UNF threads due to the limited size, as illustrated in Fig. 3a. To facilitate secure handling during testing, a pair of custom-designed sample holders with matching internal threads was fabricated, as shown in Fig. 3b. These holders ensured that the samples could be securely mounted to the machine interface grips, providing reliable grip and alignment during the test. The assembled setup, with tensile machine clamps gripping the sample via the threaded holders, is exhibited in Fig. 3c. Subsize specimens were used due to the limited availability of the GRX-810 material. This miniature threaded specimen configuration follows ASTM-adapted subsize tensile testing practice for limited material availability and has been widely used for AM-fabricated small-volume samples. Special attention was given to thread alignment and grip fixation through custom-designed holders to minimize stress concentration and misalignment effects during testing. For each condition, three specimens were tested to ensure repeatability of the tensile results, and the tensile properties reported in Table 5 are presented as mean ± standard deviation.

### Material characterization

Scanning Electron Microscopy (SEM) and Energy Dispersive Spectroscopy (EDS) were performed on the ThermoFisher Helio G5 Xe PFIB/SEM system. The EDS mapping was performed at 20 kV and 3.2 nA with an EDS detector on the same PFIB/SEM system. MicroCT was performed at a Heliscan MicroCT (Mark II) system with tungsten filament. The working voltage and current are 140 KV and 55 µA.

## Result and discussion

### Thermal diffusivity and conductivity measurements for GRX-810 with different yttria contents

#### The expectations and challenges

The yttria contents in the eight groups of L-PBF prepared GRX-810 samples vary from 0.3% to 1%, Table 2. Prior literature reported thermal diffusivity value for yttria powder is 6.52 mm^2^/s [34], which was carried out using a photoacoustic based spectroscopy method. A standard comparison of thermal diffusivity values for Nickel-based alloys (Inconel 718 and Inconel 625) range from 2.5 mm^2^/s [34] to 4.0 mm^2^/s [34] at room temperature. By measuring thermal diffusivity, specific heat, and density, thermal conductivity can be calculated using Eq. 2. For a composite of simple mixture, the effective thermal conductivity for a mixture without thermal resistance between the two different phases can be estimated using the following equation, Eq. 3

3 $${\mathrm{k}}_{{\mathrm{e}}} = {\text{ v}}_{{\mathrm{1}}} \times {\text{ k}}_{{\mathrm{1}}} \, + \,{\mathrm{v}}_{{\mathrm{2}}} \times {\text{ k}}_{{\mathrm{2}}}$$

where, k_e_ is the effective thermal conductivity, v_1_ and v_2_ are the volumetric fraction of each phase, and k_1_, k_2_ are the thermal conductivity of each phase.

Yttrium and other alloying elements in GRX-810, such as titanium, can form a stable yttrium-rich phase (usually oxides) at high temperatures. Under optimized L-PBF processing conditions, the as-built GRX-810 samples can reach 99.9% of theoretical density [35]. Given the near-full density achieved and the additional HIP treatment, Eq. 3 can reasonably assume negligible interfacial thermal resistance between the yttria particles and the GRX-810 matrix in the HIP samples. For L-PBF NHT samples, as long as a high 99.9% theoretical density was achieved, the deviation from the zero thermal resistance assumption remains small. As the yttria contents vary from 0.3% to 1.0% (Table 2), the differences in thermal properties among the GRX-810 samples are expected to be small, Eq. 3. Based on the Netzsch LFA 467 specification, the measurement uncertainty for thermal diffusivity is 3% and the measurement uncertainty for thermal conductivity is 5%. The high uncertainty associated with thermal conductivity is mainly due to the uncertainties associated with specific heat measurements. The specific heat (Cp) of samples was determined by the Netzsch LFA 467 HT apparatus via a comparison measurement with a reference sample. Under the assumption that the flash pulse energy absorbed by the sample and the energy absorbed by the reference are the same, with known specific heat value of the reference, specific heat of the sample can be calculated based on the temperature changes, Eq. 4

4 $$\:\begin{array}{c}{C}_{pr}\cdot\:{m}_{r}\cdot\:{\varDelta\:T}_{r}={C}_{pt}\cdot\:{m}_{t}\cdot\:{\varDelta\:T}_{t}\end{array}$$

where * m* is the mass of the sample, $\:\varDelta\:T$ is the maximum temperature rise of the sample during individual flashlight shot; Subscripts * r* and * t* indicate the reference and tested samples, respectively. With the already known $\:{C}_{p}$ of the reference sample, $\:{C}_{p}$ of the tested samples can be determined. The uncertainty of specific heat measurement using Netzsch LFA 467 HT is stated to be +/− 5%. Equation 4 assumes the flash pulse energy delivered to the sample and the energy delivered to the reference coupon are the same. This assumption requires an identical surface emissivity for both the GRX-810 samples and the reference sample to absorb the same amount of radiation energy. Although the surfaces of all testing samples were coated with graphite to achieve a similar emissivity value, due to uncertainties associated with the spray coating process, the conditions of the graphite spray, the conditions of residual oxygen, etc., the surface emissivity was observed to vary from batch to batch, specifically at elevated temperatures. Therefore, the equal energy assumption made in Eq. 4 may have large uncertainties.

#### Uncertainty analysis

For each set of NHT and HIP processed L-PBF GRX-810 samples, three samples were prepared. Over the testing temperature range (25 °C to 900 °C), at each testing temperature, three measurements were carried out on each sample. After cooling down to room temperature, the measurements were then repeated to check consistency.

For each NHT and HIP set, the specific heat measurements were processed to find standard deviation at each measurement temperature. Then the standard deviations were normalized by the mean values to generate an uncertainty index for each set, Table 3. The values match the specified equipment uncertainty of 5%. To determine conclusions, the data sets with high accuracy were used, namely Sets 3,4,6,7 for the NHT samples and Sets 2,4,5,6,7,8 for the HIP samples.

**Table 3:** Uncertainties with specific heat measurements

NHT yttria content (wt%)	Uncertainty (%)	HIP yttria content (wt%)	Uncertainty (%)
1	No statistical data	1	3.99
0.9	4.64	0.9	2.1
0.7	3.57	0.7	4.31
0.5	3.51	0.5	2.8
0.3	5.35	0.3	2.4
0.45	1.76	0.45	2.24
0.4	1.51	0.4	1.42
0.35	5.43	0.35	2.15

#### Correction for thermal expansions

The linear thermal expansion coefficient is described by the following equation,

5 $$\:\frac{\mathrm{d}L}{L}={\alpha}_{L}\mathrm{d}t$$

where * L*, $\:{\alpha}_{L}$, and * t* indicate the length of the material, linear coefficients of thermal expansion, and temperature, respectively. Meanwhile, coefficient of volumetric thermal expansion ($\:{\alpha}_{V}$) is described by formula (6).

6 $$\:\frac{\mathrm{d}V}{V}={\alpha}_{V}\mathrm{d}t$$

where * V* is the volume of the material. For isotropic materials, the relation between $\:{\alpha}_{V}$ and $\:{\alpha}_{L}$ is shown below,

7 $$\:{\alpha}_{V}=3{\alpha}_{L}$$

With the expressions of linear coefficients of thermal expansion of the alloys, by integral of formula (5) and formula (6), the linear change and volumetric change of the alloys as a function of temperature can be calculated, shown in formula (8) and (9), respectively.

8 $$\:\frac{{L}_{t}}{{L}_{0}}={e}^{\int\limits_{{t}_{o}}^{t}{\alpha}_{L}\left(t\right)\mathrm{d}t}$$

9 $$\:\frac{{V}_{t}}{{V}_{0}}={e}^{\int\limits_{{t}_{o}}^{t}{\alpha}_{V}\left(t\right)\mathrm{d}t}$$

where $\:{L}_{t}$, $\:{V}_{t}$ are the length and volume of the material at temperature * t*; $\:{L}_{0}$, $\:{V}_{0}$ indicate the length and volume of the material at temperature $\:{t}_{o}$ (room temperature, 25 ℃); $\:{\alpha}_{L}\left(t\right)$ and $\:{\alpha}_{V}\left(t\right)$ represent the temperature dependent linear thermal expansion coefficient and volumetric thermal expansion coefficient, respectively.

Two parameters need to be modified with the linear and volumetric thermal expansion, namely density and thermal diffusivity. The temperature dependent density ($\:{\rho\:}_{t}$) is described below in formula (10), where $\:{\rho\:}_{0}$ is the density at room temperature (25 ℃).

10 $$\rho _{t} = {{\rho _{0} } \mathord{\left/ {\vphantom {{\rho _{0} } {\left( {\frac{{V_{t} }}{{V_{0} }}} \right)}}} \right. \kern-\nulldelimiterspace} {\left( {\frac{{V_{t} }}{{V_{0} }}} \right)}}$$

During the thermal property test, constant thickness of the tested sample was used. Therefore, with formula (8), the temperature dependent thermal diffusivity ($\:{a}_{t}$) can be modified by the following formula.

11 $$\:{a}_{t}={a}_{t,tested}\cdot\:{\left(\frac{{L}_{t}}{{L}_{0}}\right)}^{2}$$

With the formulas above, all the thermal property data can be modified with coefficients of thermal expansion. Table 4 lists thermal expansion data vs. Temperature for the HIP and NHT L-PBF GRX-810 samples with a fixed 1% yttria content. Corrections are made to thermal diffusivity and conductivity data listed in the next section.

**Table 4:** Thermal expansion data vs. Temperature in HIP and NHT L-PBF GRX-810

Temperature (°C)	HIP CTE (10^−6^ m/m/°C)	NHT CTE (10^−6^ m/m/°C)
100	11.8	10.6
200	12.9	12.3
300	13.5	13.0
400	13.9	13.5
500	14.3	13.9
600	14.7	13.9
700	15.4	14.4
800	16.0	15.0
900	16.5	15.5
1000	17.2	16.1

#### Thermal diffusivity and conductivity data

**Fig. 4:**
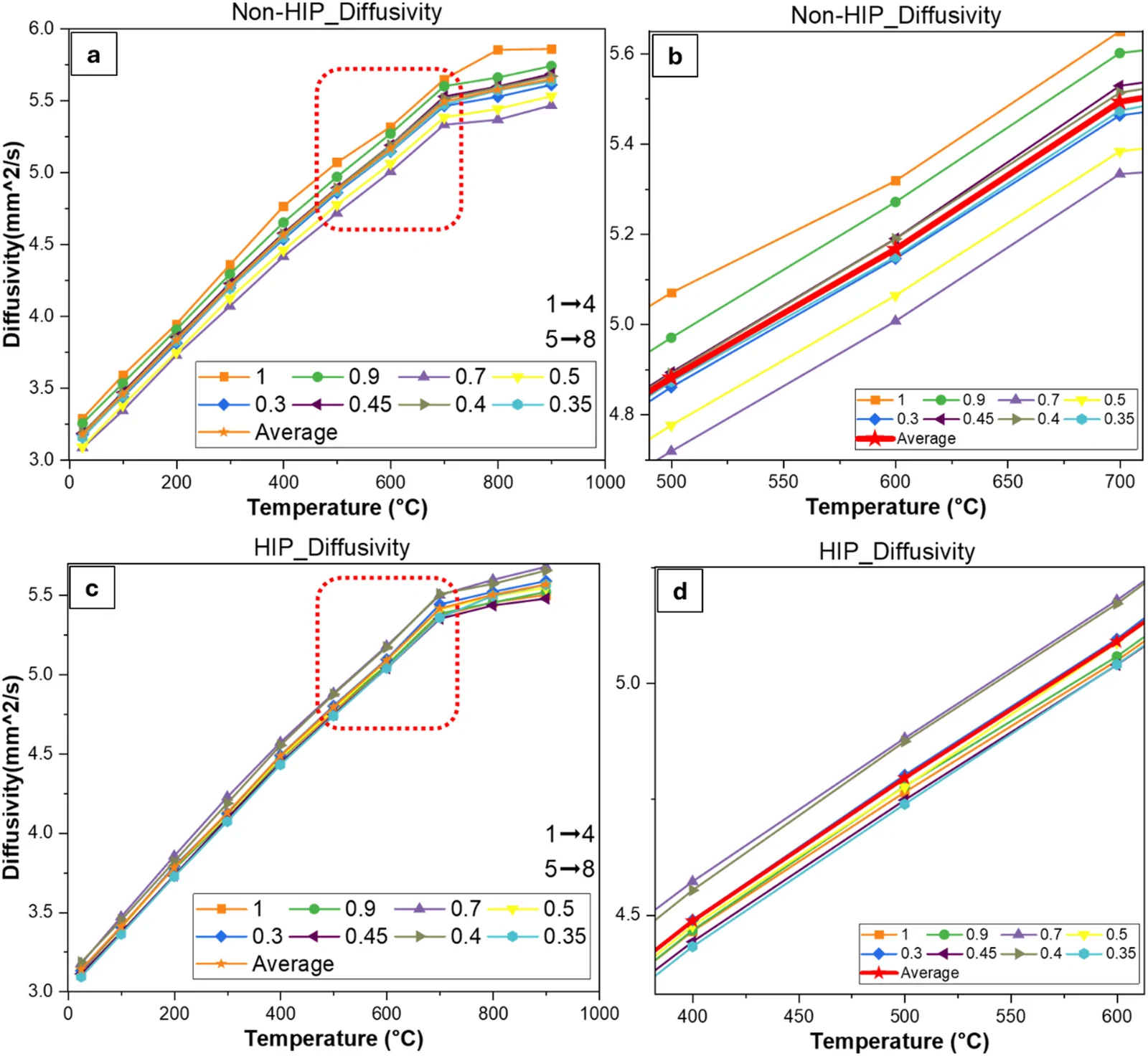
Thermal diffusivity of **a**–**b** NHT GRX-810 sample and **c**–**d** HIP GRX-810 samples

As shown in Fig. 4, the thermal diffusivity of GRX-810 alloys increases monotonically with temperature for both NHT and HIP-processed conditions, with substantial overlap among different yttria contents. To quantitatively assess the significance of these variations, 600 °C is selected as a representative temperature, as it lies within the mid–high temperature regime relevant to service conditions and captures the characteristic behavior observed across the entire temperature range. At this temperature, the relative variation in thermal diffusivity across different yttria contents is ~ 2.0% for NHT samples and further reduced to ~ 1.1–1.5% after HIP, both of which remain within the ~ 3% instrumental uncertainty of the measurement technique. A comparison between low yttria contents (< 0.5 wt%) and high yttria contents (> 0.5 wt%) shows that increasing yttria by ~ 0.2 wt% leads to an average reduction in thermal diffusivity of ~ 2% in the NHT condition. Thus, the observed slight reduction in thermal diffusivity with an increasing yttria content was based on the evaluation of the highest accuracy data sets (3,4,6,7 for the NHT). The corresponding variation after HIP is below 1% and not statistically distinguishable from experimental uncertainty. These results indicate that yttria content has a small to negligible influence on thermal diffusivity, particularly after HIP processing.

**Fig. 5:**
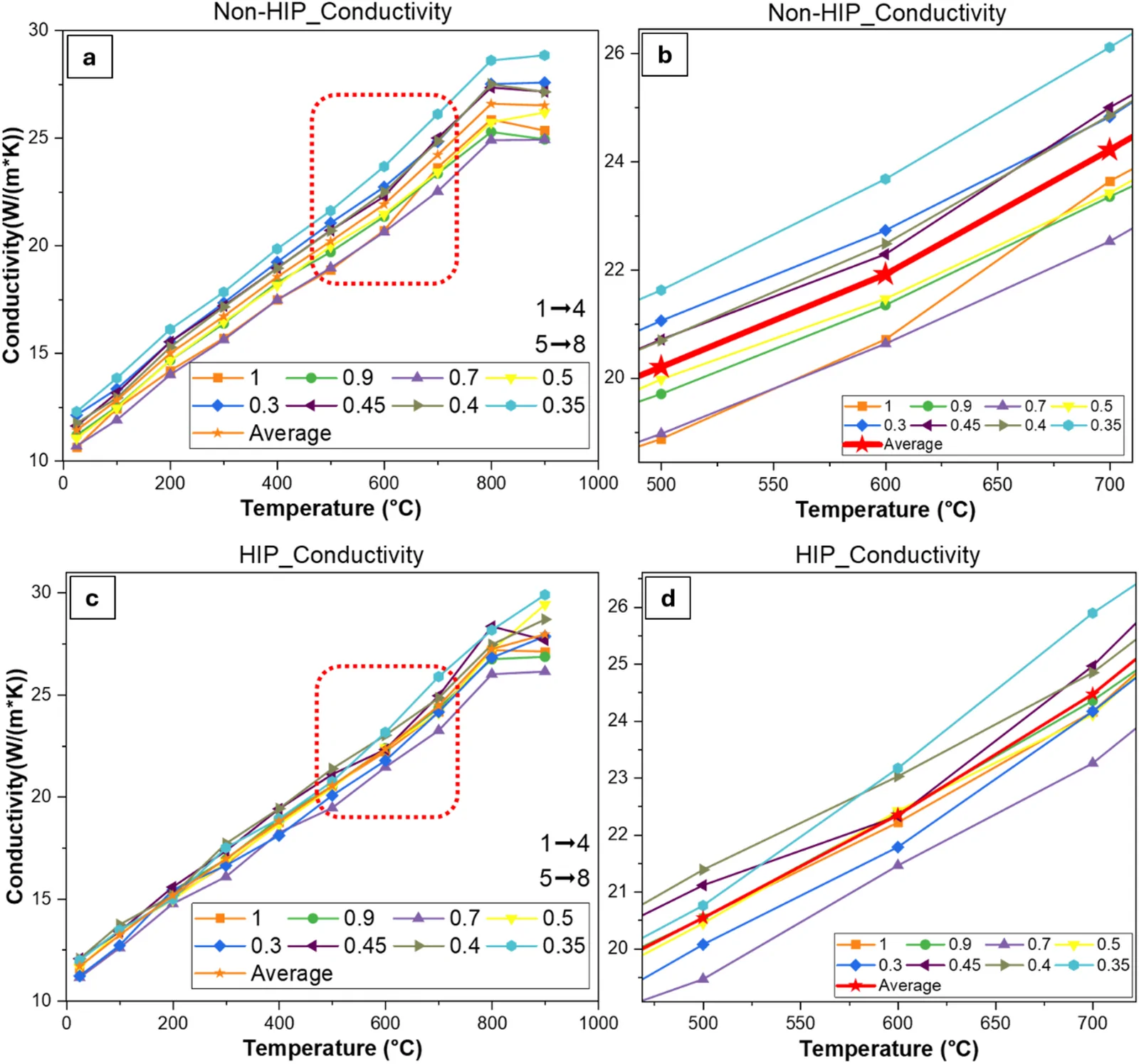
Thermal conductivity of **a**–**b** NHT GRX-810 samples and **c**–**d** HIP GRX-810 samples

In contrast, Fig. 5 reveals a more pronounced dependence of thermal conductivity on yttria content, especially at elevated temperatures. Using 600 °C as a representative reference point, the relative standard deviation of thermal conductivity reaches ~ 4.8% for NHT samples, which is close to the ~ 5% instrumental uncertainty. Therefore, the conductivity differences should be interpreted conservatively as a modest but physically consistent trend rather than a strong composition dependence. At the same temperature, HIP processing reduces this variation to ~ 2.5%, within the instrumental uncertainty limit. This behavior is consistent with the trends observed throughout the temperature range. At higher temperatures, such as 900 °C, the separation becomes more evident, where a low-yttria sample (#7, 0.40 wt% Y_2_O_3_) exhibits a thermal conductivity of ~ 27.1 W m^−1^ K^−1^ compared with ~ 24.9 W m^−1^ K^−1^ for a high-yttria sample (#3, 0.70 wt% Y_2_O_3_), corresponding to an ~ 8% difference. Overall, while yttria content introduces measurable variations in thermal conductivity in the NHT state, the thermal transport behavior should not be attributed to yttria content alone. Because porosity content, grain boundary density, and dispersoid distribution were not quantitatively measured for each composition before and after HIP in the present study, these factors are discussed here as possible microstructural contributors based on established thermal-transport mechanisms rather than as directly quantified variables. Therefore, the observed thermal conductivity variation should not be attributed to yttria content alone. Instead, nominal yttria content, defect population, grain-boundary scattering, and dispersoid distribution may collectively influence electron and phonon transport in L-PBF GRX-810 alloys, as reported for AM Ni-based superalloys and oxide-dispersion-strengthened alloys [1, 12, 24, 26, 35]. The reduced conductivity scatter observed after HIP is consistent with the possible effects of defect reduction and improved microstructural homogeneity commonly associated with HIP treatment in L-PBF Ni-based alloys [27–29]. However, because quantitative porosity, grain-boundary density, and dispersoid-distribution measurements were not performed for each composition before and after HIP, the relative contributions of these factors cannot be independently separated in the present study. Future work will combine quantitative porosity analysis, EBSD-based grain-boundary characterization, and high-resolution oxide/dispersoid mapping to determine the individual contributions of these microstructural factors to thermal transport in GRX-810.

To further quantify the temperature dependence, cubic polynomial functions were employed to fit both thermal diffusivity and thermal conductivity data over the temperature range of 25–900 °C. These fitted functions are intended only as empirical descriptions of the experimental data to provide continuous interpolation and facilitate quantitative comparison between different compositions and processing conditions, rather than as mechanistic models of thermal transport behavior. As shown in the Eqs. 12–14, the fitted curves show excellent agreement with the experimental measurements (R² ≈ 0.99), providing a continuous description of thermal transport behavior. Notably, the fitted diffusivity curves for different yttria contents largely overlap, consistent with the uncertainty analysis indicating negligible composition dependence. In contrast, the fitted conductivity curves display clearer separation for the NHT condition, reinforcing that the observed conductivity variations represent a small but physically consistent effect of composition and processing. These empirical fits therefore summarize the experimental observations and facilitate quantitative comparison between HIP and non-HIP samples, but do not provide independent mechanistic validation.

Non-HIP

12 $$\:\begin{array}{c}\alpha\left(T\right)=\:-2.7147{T}^{3}+1.8861{T}^{2}+3.3375T+3.1037\end{array}$$

13 $$\:\begin{array}{c}\kappa\left(T\right)=\:-9.3152{T}^{3}+12.3369{T}^{2}+12.8474T+11.1657\end{array}$$

HIP

14 $$\:\begin{array}{c}\alpha\left(T\right)=\:-2.9077{T}^{3}+2.2699{T}^{2}+3.0939T+3.0739\end{array}$$

15 $$\:\begin{array}{c}\kappa\left(T\right)=\:-1.8961{T}^{3}+3.1259{T}^{2}+17.5652T+11.3917\end{array}$$

where *T* is temperature in K/1000.

### Tensile test of GRX-810

Tensile testing was conducted at a constant strain rate of 0.4 mm/min and at ambient temperature. A small 6 mm mechanical extensometer was implemented for strain measurements. Due to the small spring configuration of the miniature mechanical extensometer, occasional sudden slippage was observed during a few tests. However, this occurred only during part of the deformation process and did not affect the final elongation values or the overall strength–ductility trends reported in this study.

**Fig. 6:**
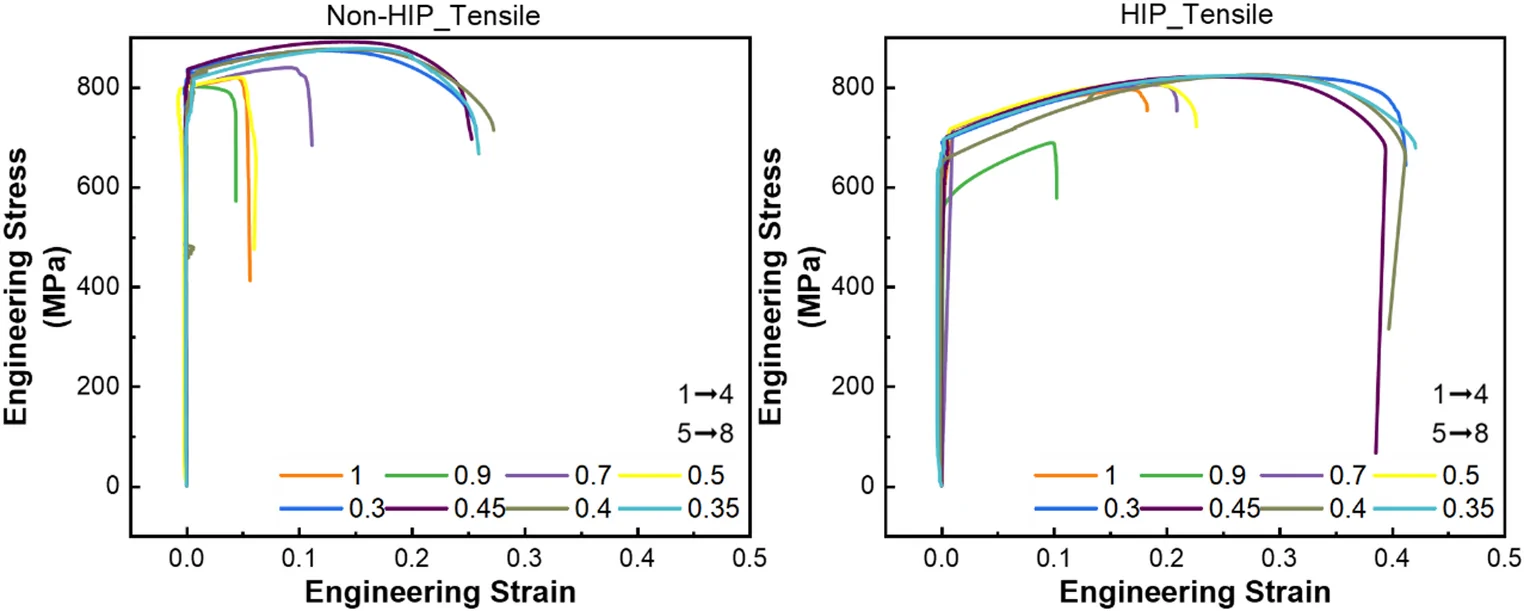
Engineering stress-strain curves of NHT GRX-810 samples and HIP GRX-810 samples

Figure 6 presents the tensile stress–strain responses of GRX-810 samples (along the L-PBF horizontal direction) with different yttria contents in the NHT and HIP-processed conditions, while the corresponding yield strength, ultimate tensile strength, and elongation are summarized in Table 5. Clear composition- and processing-dependent trends are observed.

**Table 5:** Tensile test results for HIP and NHT AM GRX-810

Sample	Y_2_O_3_ (wt%)	Yield strength (MPa)		Ultimate tensile strength (MPa)		Elongation (%)	
		Non-HIP	HIP	Non-HIP	HIP	Non-HIP	HIP
1	1	733 ± 19	720 ± 11	820 ± 21	796 ± 10	5 ± 2	18 ± 2
2	0.9	732 ± 26	570 ± 12	801 ± 19	689 ± 13	3 ± 3	10 ± 1
3	0.7	741 ± 24	720 ± 15	840 ± 23	810 ± 14	11 ± 3	21 ± 3
4	0.5	797 ± 18	716 ± 14	820 ± 20	809 ± 9	6 ± 4	23 ± 2
5	0.3	813 ± 28	692 ± 9	874 ± 22	824 ± 11	26 ± 5	41 ± 2
6	0.45	837 ± 23	700 ± 10	892 ± 28	822 ± 16	25 ± 4	39 ± 2
7	0.4	817 ± 19	660 ± 8	877 ± 19	825 ± 18	27 ± 5	41 ± 3
8	0.35	813 ± 22	700 ± 11	878 ± 26	824 ± 17	26 ± 4	42 ± 2

For the NHT samples, lower yttria content generally leads to high yield strength and high ductility. All samples exhibit yield strengths in the range of 732–837 MPa, with alloys containing higher yttria contents (> 0.5 wt%) showing consistently lower yield strengths of ~ 730–797 MPa, and limited elongation of only 4–11%. In contrast, the yield strengths for lower-yttria alloys (< 0.5 wt%) are high (813–837 MPa) while exhibiting substantially improved ductility, with elongations increasing to 25–27%. Ultimate tensile strength (UTS) follows a similar trend: high-yttria samples exhibit UTS values of 801–840 MPa, whereas low-yttria samples reach higher UTS values of 874–892 MPa, indicating a more favorable strength–ductility balance at reduced yttria contents.

HIP processing induces a pronounced shift in tensile behavior across all compositions. In addition to possible pore closure and microcrack healing, HIP-induced grain coarsening, recrystallization, reduced dislocation density, and residual stress relaxation may also contribute to the observed mechanical response under the present processing condition (1163 °C, 3 h, 104 MPa), as commonly reported for HIP-treated L-PBF Ni-based alloys [27–29]. However, direct quantification of these microstructural changes is beyond the scope of the present study and will be addressed in an ongoing microstructure-focused follow-up investigation. Although the yield strength is reduced after HIP, typically by 20–160 MPa depending on yttria content, the elongation increases dramatically. For high-yttria alloys (> 0.5 wt%), HIP increases elongation from 4 to 11% to 10–23%, while low-yttria alloys (< 0.5 wt%) exhibit a much more significant enhancement, reaching elongations of 39–42%. Notably, samples with 0.30–0.40 wt% Y_2_O_3_ (samples #5, #7, and #8) retain relatively high ultimate tensile strengths of ~ 822–825 MPa after HIP while achieving elongations exceeding 40%, highlighting the strong synergy between reduced yttria content and HIP treatment in improving the mechanical properties. Therefore, the improved ductility and reduced yield strength after HIP are interpreted as a combined consequence of defect-related improvement and thermally induced microstructural recovery, rather than crack elimination alone. This interpretation is consistent with the present tensile trends and prior HIP studies, while detailed verification of pore evolution, crack healing, grain coarsening, recrystallization, and residual stress relaxation will be presented in a subsequent study.

These tensile results demonstrate that yttria content plays a critical role in governing the strength–ductility trade-off of GRX-810 alloys. Higher yttria additions promote strength (specifically at high temperatures) but severely limit ductility, whereas lower yttria contents enable both high strength and exceptional plasticity. HIP processing further amplifies this effect by significantly enhancing ductility while maintaining competitive strength levels, making low-yttria GRX-810 alloys (< 0.5 wt% Y_2_O_3_) particularly attractive for applications requiring a balanced combination of strength and deformation capability.

A similar trend was also reported by NASA researchers [1], where HIP-treated GRX-810 samples exhibit consistently lower ultimate tensile strength than their as-built counterparts at low temperatures, but converge toward comparable strength levels as temperature increases [1]. Notably, the difference between HIP and as-built conditions diminishes above ~ 600 °C, indicating that defect elimination and microstructural homogenization induced by HIP primarily influence room- and intermediate-temperature mechanical behavior [1]. This consistency between the present results and NASA’s high-temperature tensile data further supports the role of HIP in redistributing the strength–ductility balance without compromising high-temperature load-bearing capability.

To better understand the impact of yttria content on tensile properties, the fracture surfaces of tensile samples were examined. Tensile data in Table 5 indicate that yttria contents less than 0.45% lead to higher elongation.

**Fig. 7:**
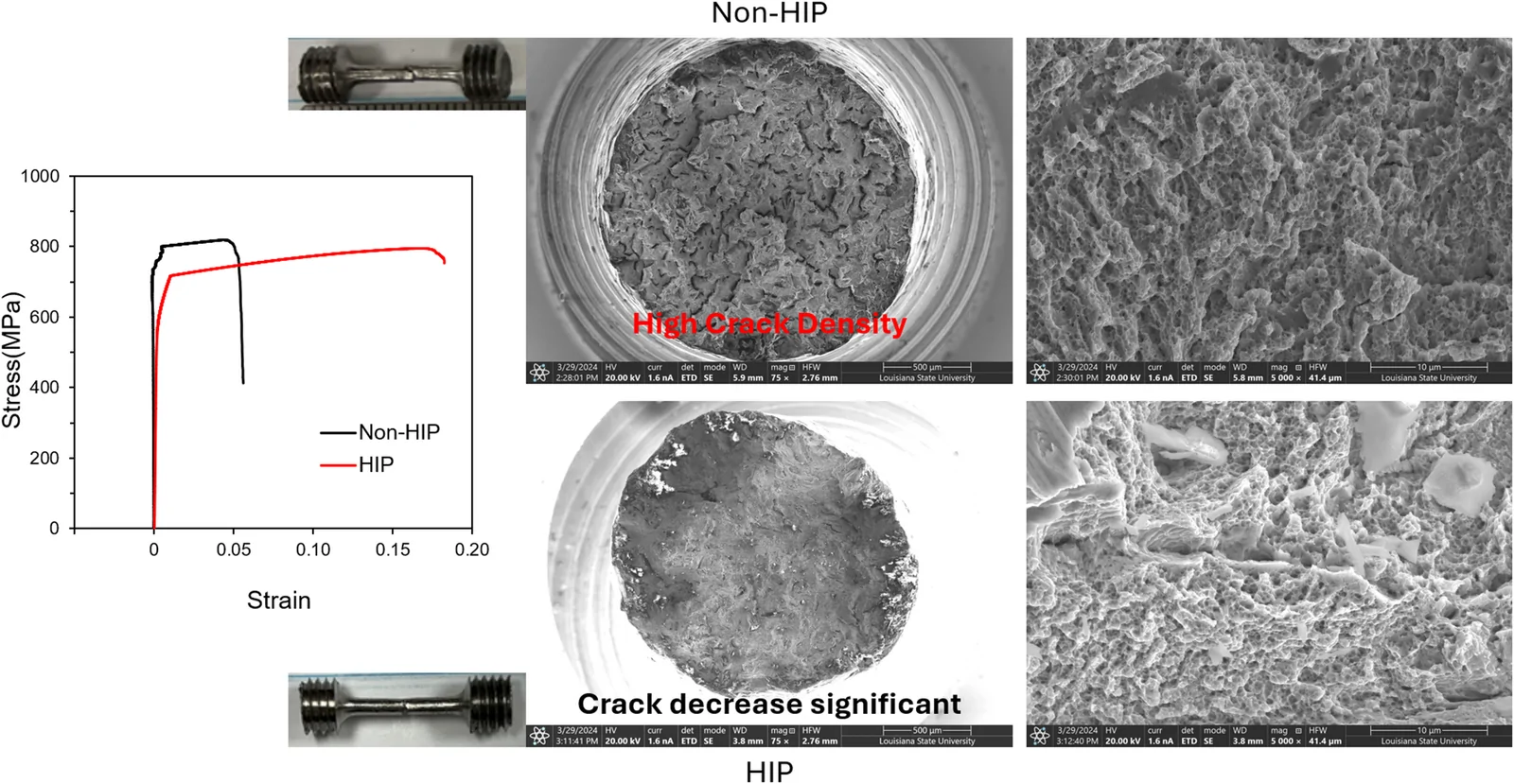
Tensile test curve of HIP and NHT GRX810-1 and the fracture surface

**Fig. 8:**
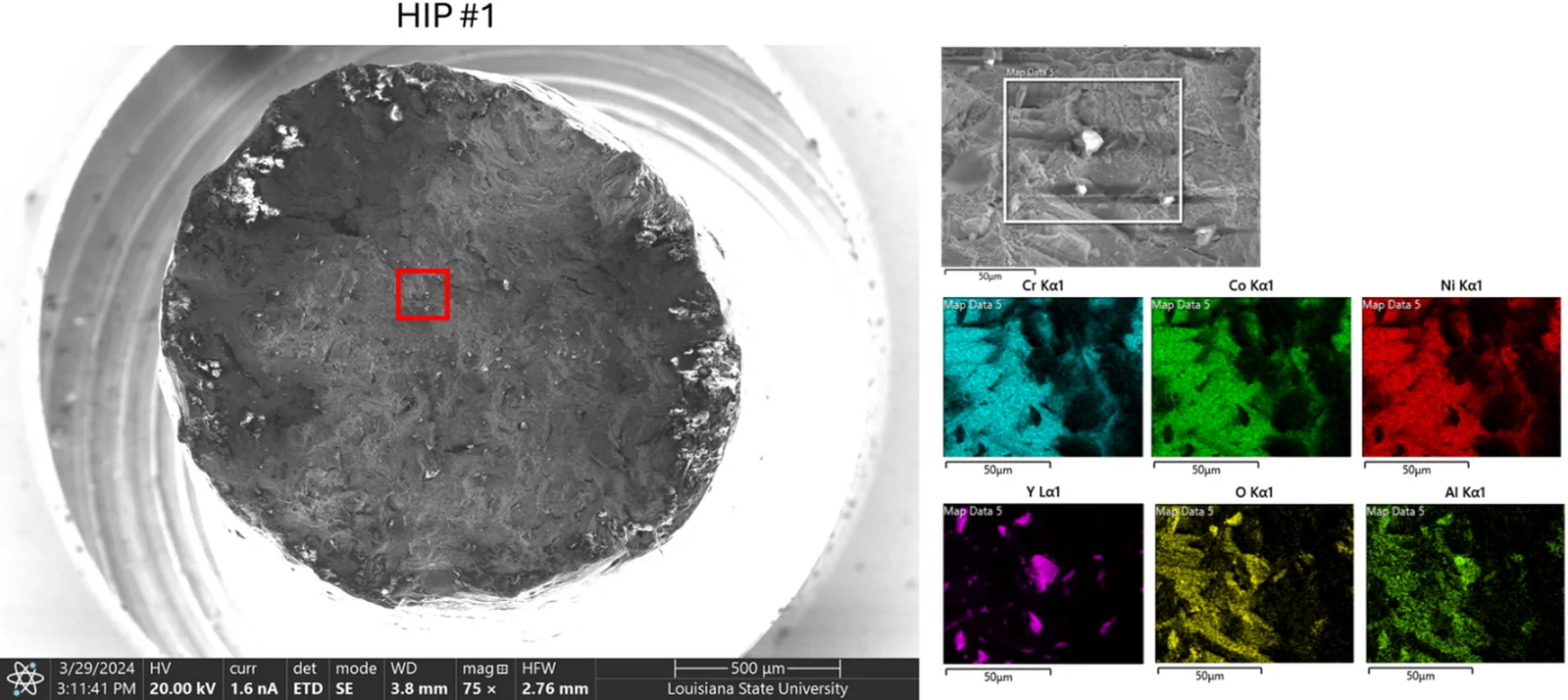
EDS mapping for the HIP GRX810-1 on the fracture surface

Figure 7 presents a direct comparison of the tensile curves of HIP and Non-HIP #1 samples, along with their respective fracture morphologies. Sample #1 has the highest yttria content (1%) among all samples. It can be observed that the fracture surface of the Non-HIP sample contains numerous cracks, which significantly reduce the sample’s ductility. After HIP treatment, these cracks disappear, leading to improved ductility. Meanwhile, compared to the NHT sample, the yield strength of the HIP sample decreases by 2.9%, while its elongation to failure increases by 260%. Figure 8 shows the EDS mapping of the HIP fracture surface, where the bright white particles are identified as yttria. These particles are primarily located at the cleavage surfaces, indicating that they contribute to increased brittleness in the matrix.

**Fig. 9:**
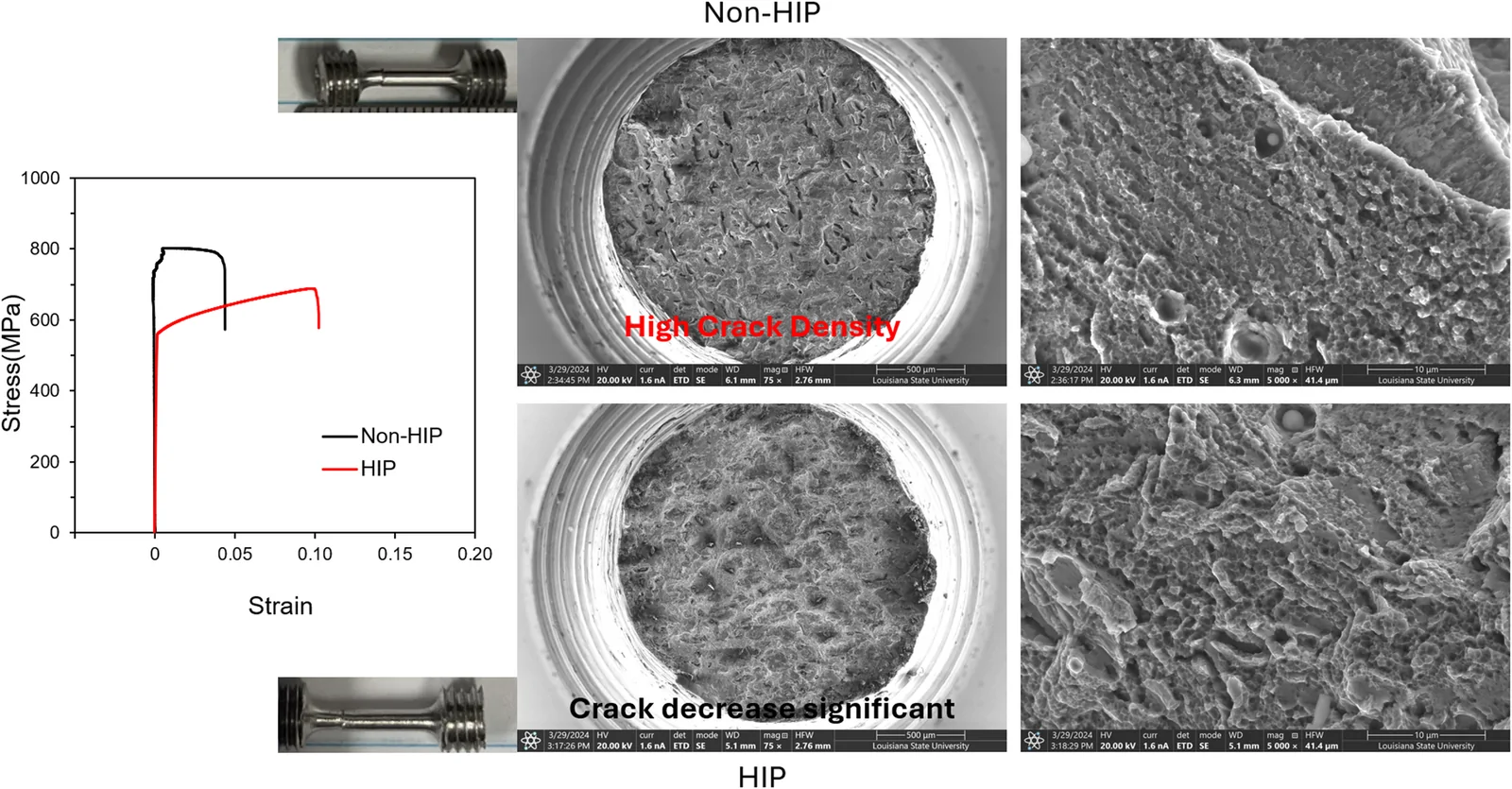
Tensile test curve of HIP and NHT GRX810-2 and the fracture surface

**Fig. 10:**
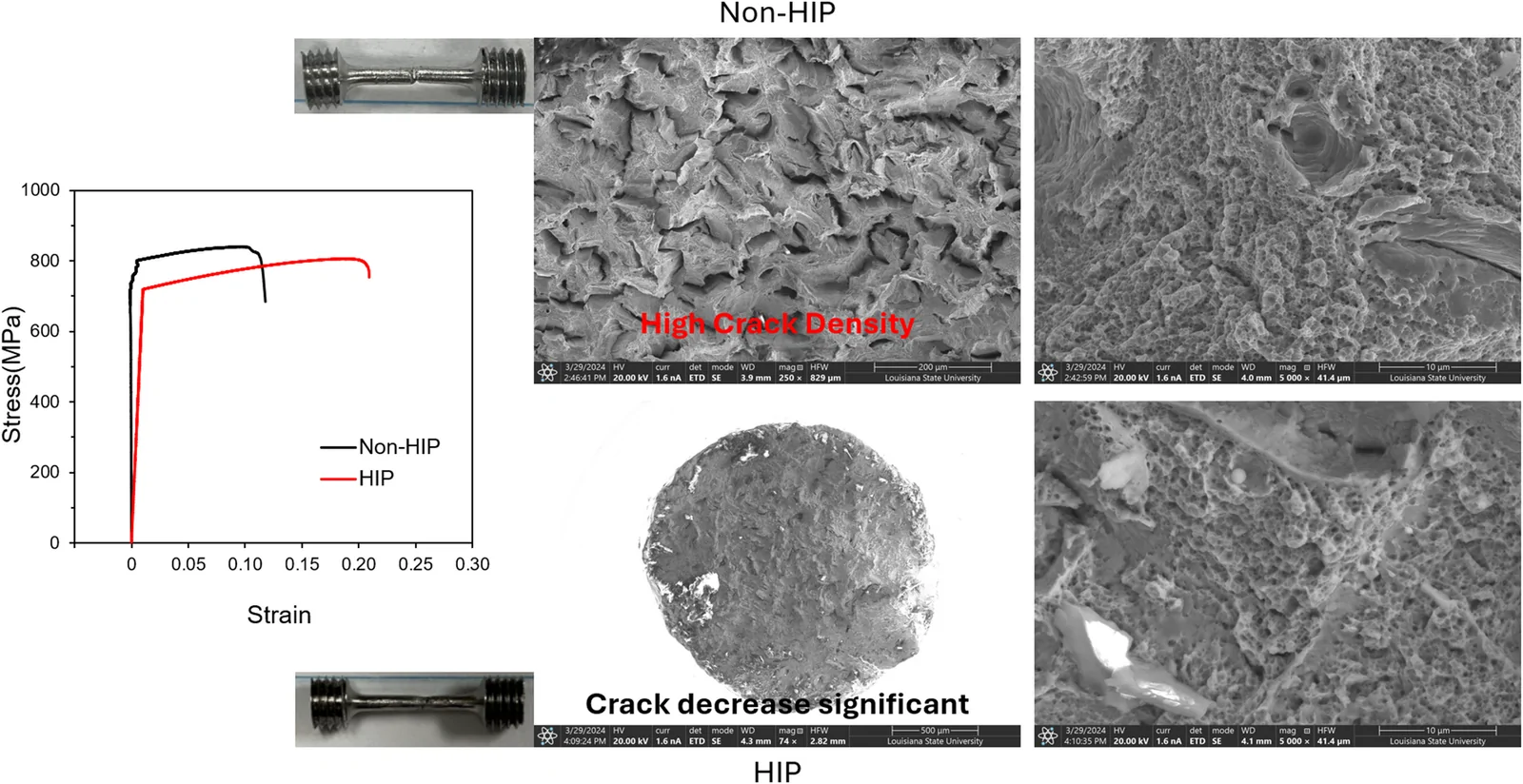
Tensile test curve of HIP and NHT GRX810-3 and the fracture surface

Sample #2 and #3 contain relatively high yttria content (0.9 and 0.7%). Figures 9 and 10 present a direct comparison of the tensile curves of these two types of samples, following with their respective fracture morphologies. It can be observed that the fracture surface of the NHT sample contains multiple cracks, drawing significantly reduction of the samples’ ductility. After HIP treatment, these cracks disappear and the tensile tests indicate an improvement of ductility. Meanwhile, compared to the NHT sample, the yield strength of the HIP sample#2 and #3 decreases by 22.1 and 2.8%, respectively, while its ductility increases by 150 and 91%.

**Fig. 11:**
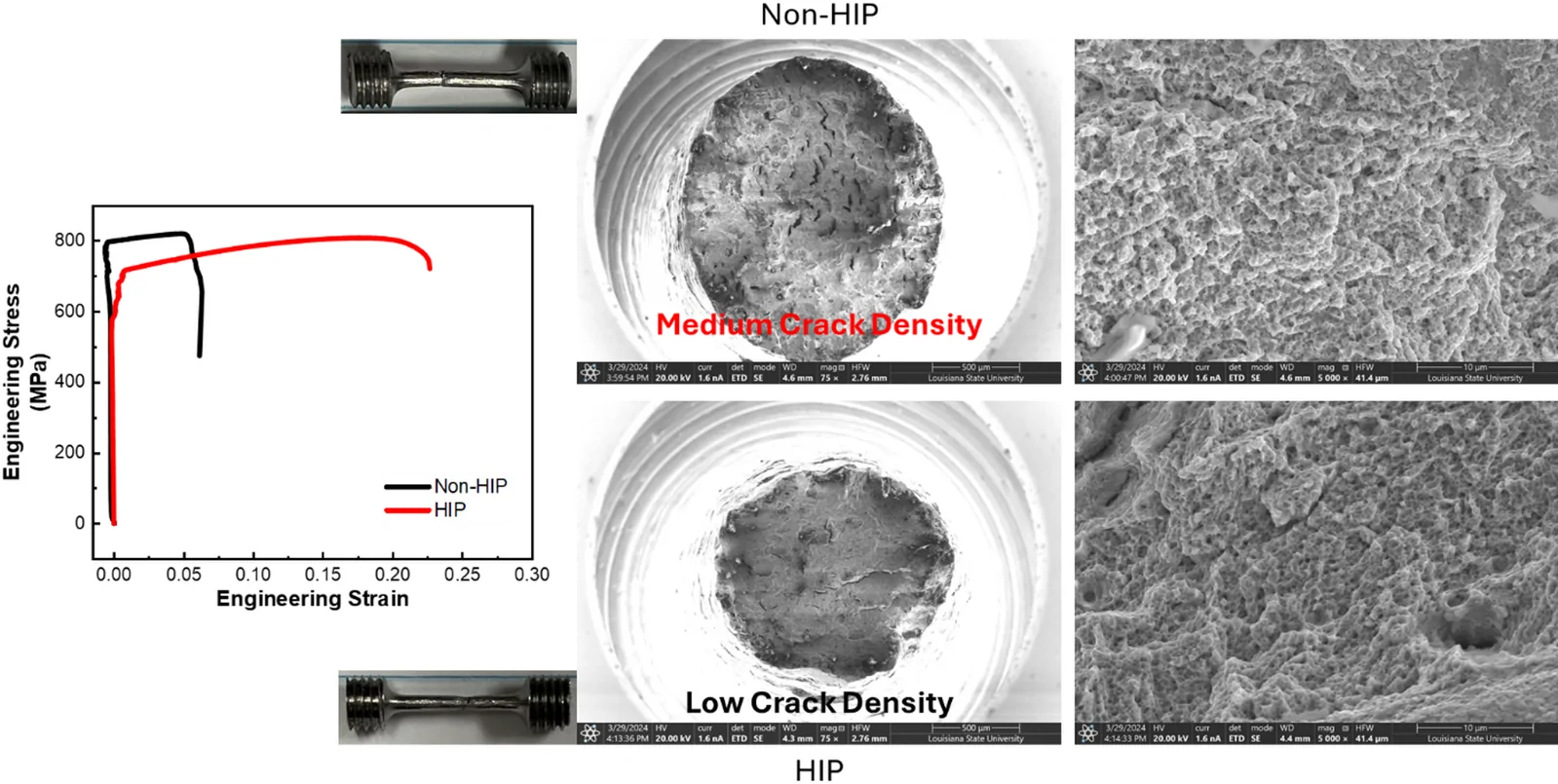
Tensile test curve of HIP and NHT GRX810-4 and the fracture surface

Sample #4 has a relatively moderate yttria content (0.5%) and the related tensile results and material characterizations are shown in Fig. 11. The SEM images show that Non-HIP sample #4 contains reduced amount of cracks (in comparison to Samples 2 & 3). The HIP treatment further reduces the number of cracks; Therefore, improve the ductility. Compared to the Non-HIP sample, the yield strength of the HIP sample #4 exhibits a 10.2% reduction, but a 283% improvement in ductility, which could be attributed to the disappearance of microcracks.

**Fig. 12:**
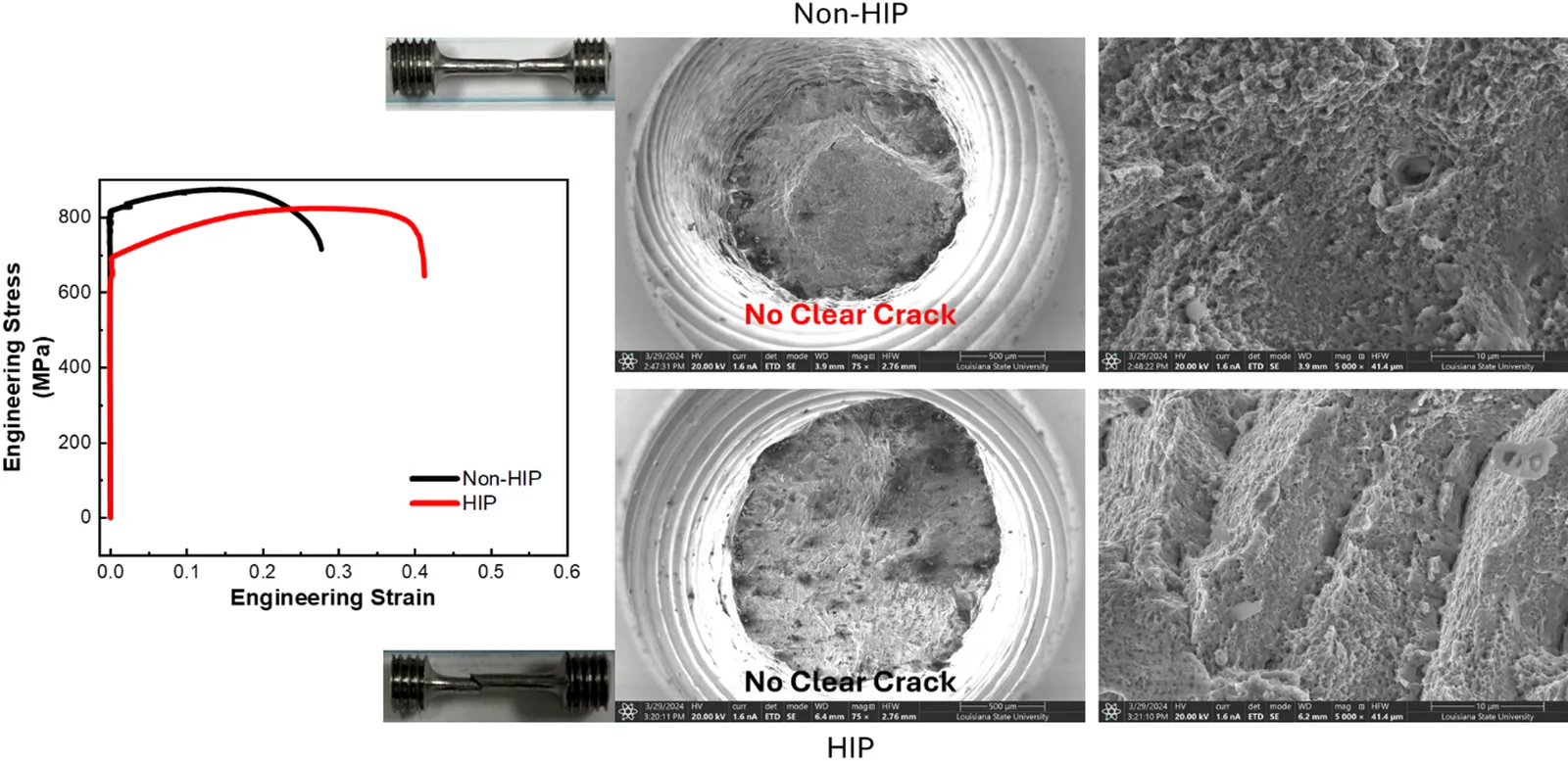
Tensile test curve of HIP and NHT GRX810-5 and the fracture surface

Sample #5 has the lowest yttria content (0.3%) and the corresponding tensile test result and fracture morphologies are exhibited in Fig. 12. No clear cracks are observed in the Non-HIP sample, and similarly, no cracks appear on the HIP treated sample. The yield strength dropped 14.9% after HIP process and the elongation at failure improved by 57.7%.

**Fig. 13:**
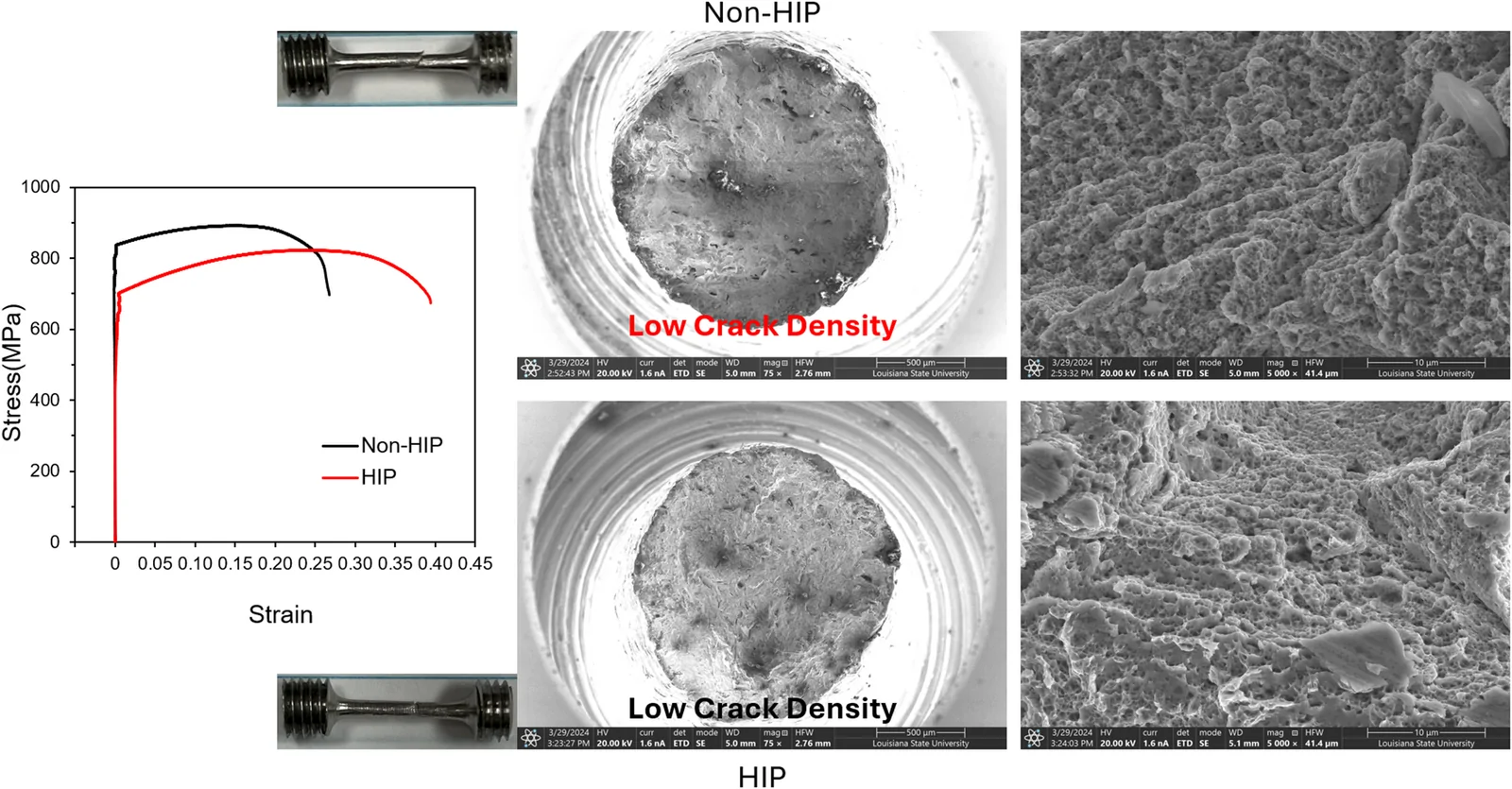
Tensile test curve of HIP and NHT GRX810-6 and the fracture surface

Figure 13 presents a direct comparison of the tensile curves of HIP and Non-HIP #6 samples, along with their respective fracture morphologies. It can be observed that the fracture surface of the Non-HIP sample contains low density cracks. After HIP treatment, the matrix still maintained low-density cracks. Compared to the Non-HIP sample, the yield strength of the HIP sample decreases by 16.4%, while its ductility increases by 56%. Overall, the ductility of Sample #6 is significantly higher than that of Sample #1.

**Fig. 14:**
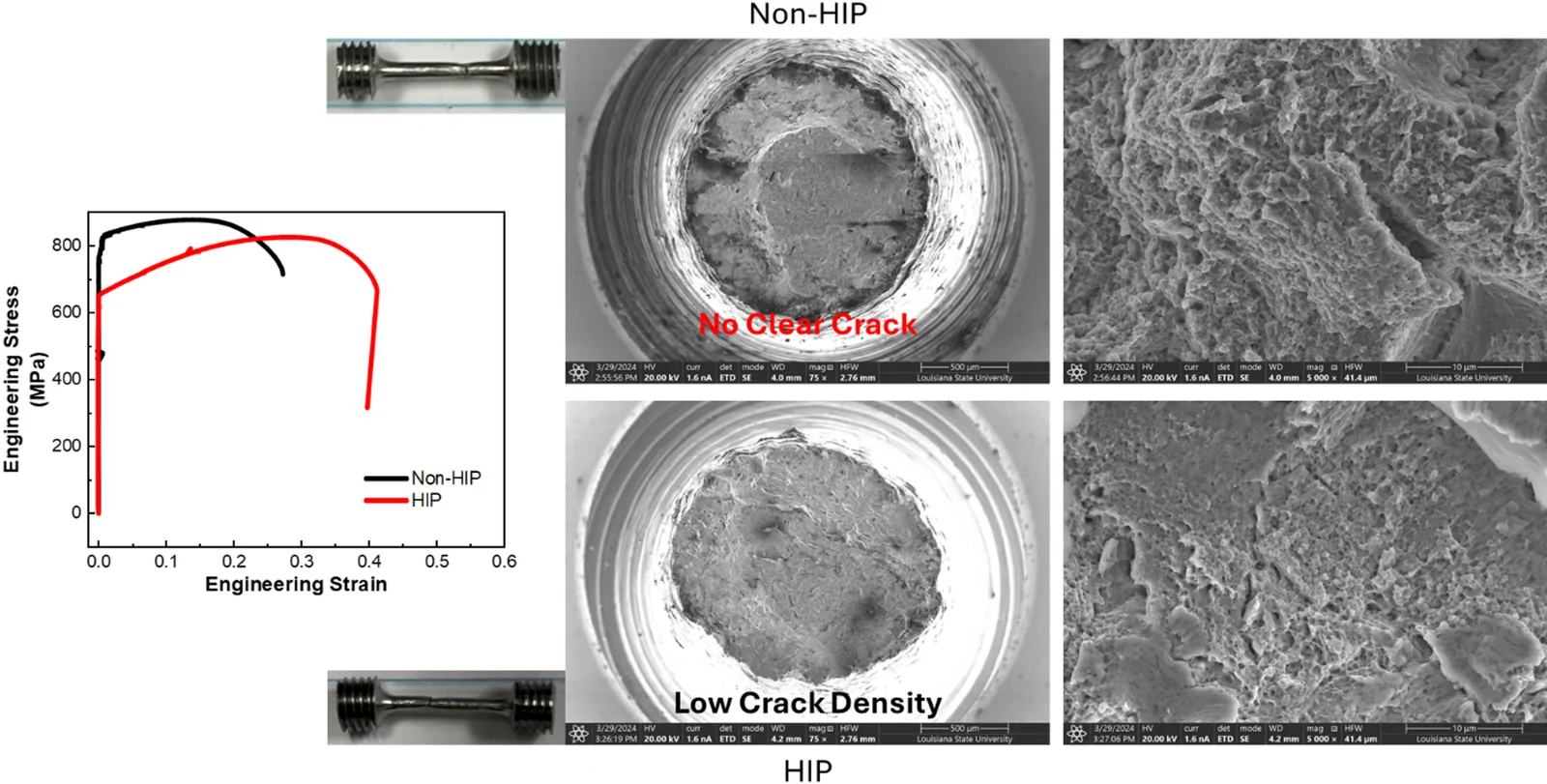
Tensile test curve of HIP and NHT GRX810-7 and the fracture surface

**Fig. 15:**
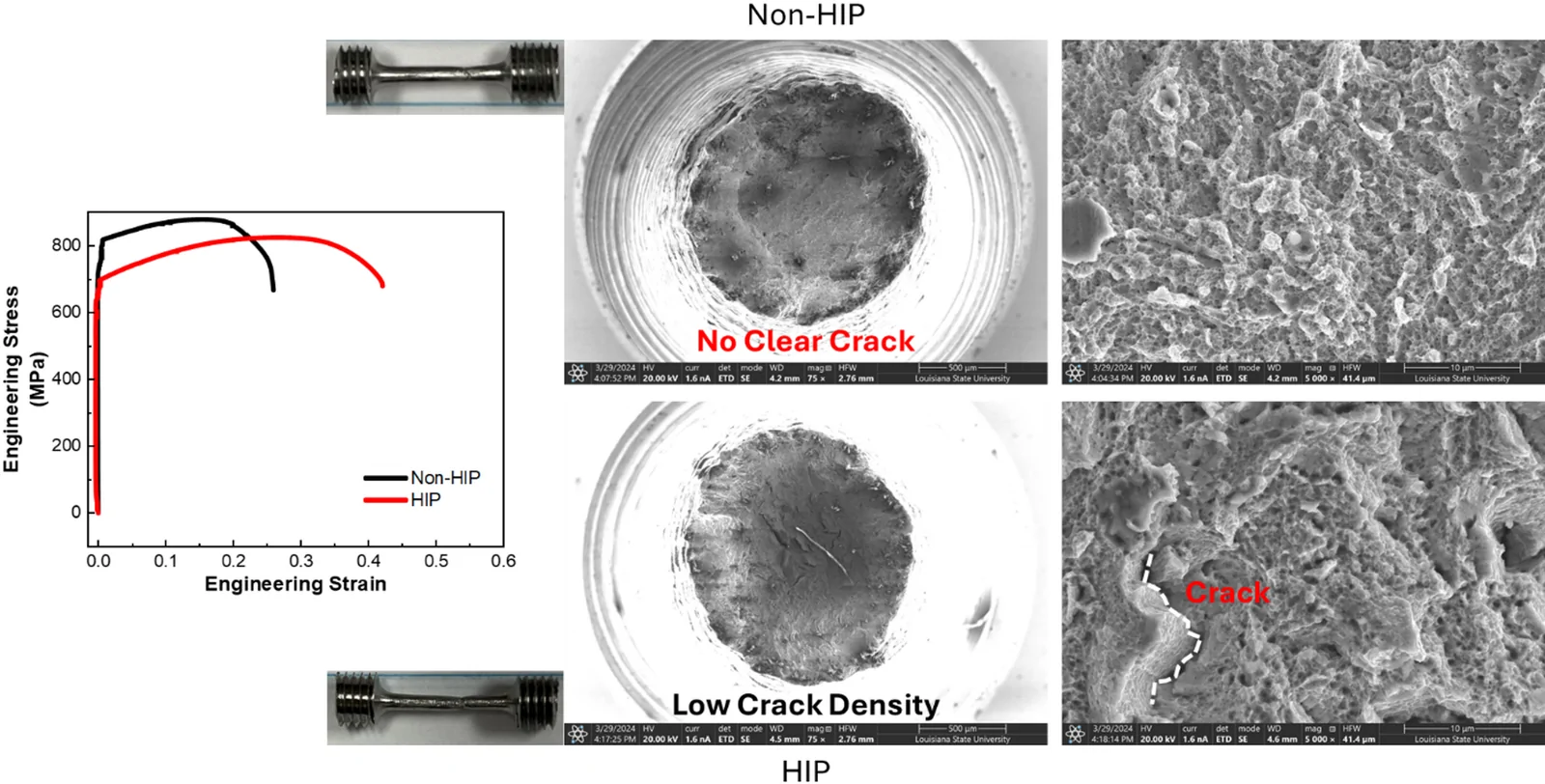
Tensile test curve of HIP and NHT GRX810-8 and the fracture surface

Samples #7 and #8 contain the relatively low yttria content (0.4% and 0.35%, respectively). Figures 14 and 15 provide a direct comparison of the tensile curves of these two samples, accompanied by their corresponding fracture morphologies. No clear cracks can be observed in Non-HIP samples, resulting in reasonably high ductility.

**Fig. 16:**
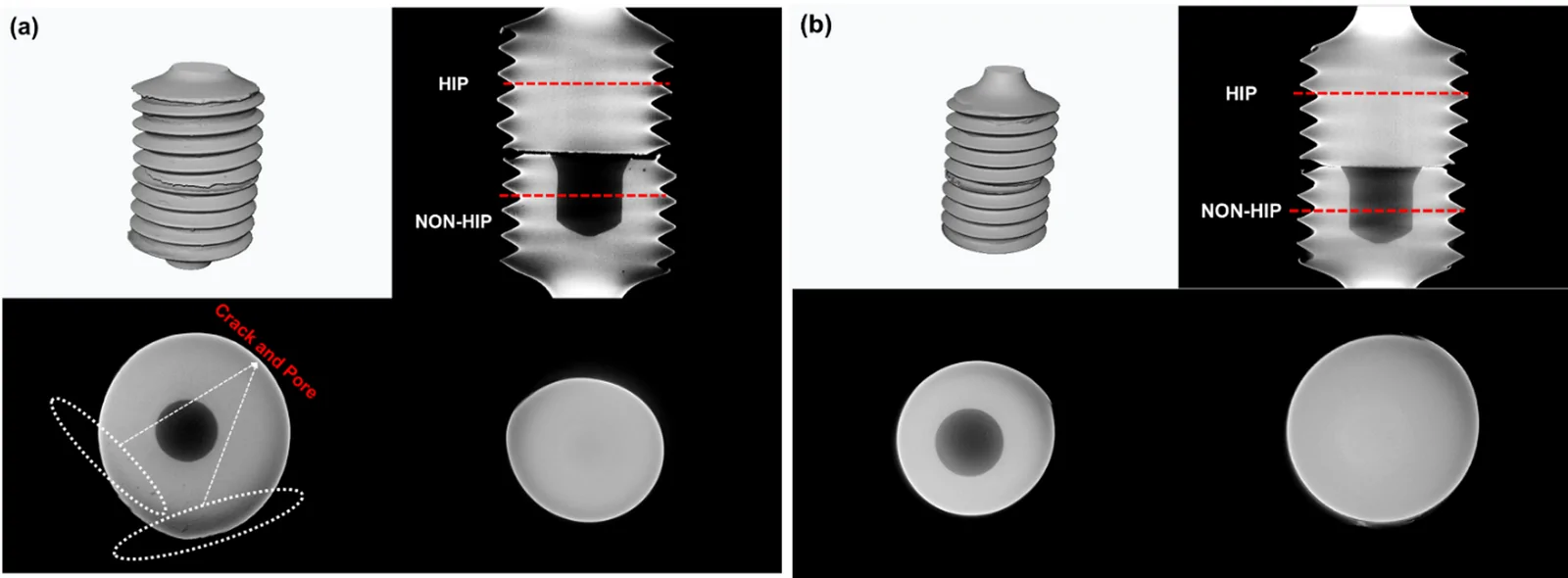
**a** MicroCT for HIP and NHT GRX-810-1 **b** MicroCT for HIP and NHT GRX-810-6

Microcracks are observed on most of the fractured surfaces, particularly in the L-PBF horizontal specimens, which are more sensitive to microcracks forming in parallel to the build direction. The reduced ductility in the high-oxide specimens is consistent with microcrack formation and possible oxide agglomeration; however, a fully quantitative fracture-mechanism analysis, including particle size distribution and interparticle spacing evaluation, is beyond the scope of the present study. Therefore, the present observations should be interpreted as evidence-consistent rather than definitive proof of the ductility reduction mechanism. Notably, this effect is no longer evident below 0.5% oxide content. To further examine whether these cracks were formed during tensile testing or in existence inside the original samples, MicroCT scanning was performed on samples that had not undergone tensile testing, as shown in Fig. 16. The results indicate that, except for the #1 Non-HIP sample, where pores were detected, no pores were found in the #1 HIP and #6 samples with the MicroCT resolution of 1 micron. This suggests that large pre-existing pores are not the dominant feature in most samples. However, due to the MicroCT resolution limit (~ 1 μm), smaller submicron defects and nanoscale pores cannot be excluded. Therefore, the observed cracks may be associated with tensile deformation, but their complete absence before testing cannot be definitively confirmed. Further research is necessary to understand the effects of both high and low yttria concentrations on phase formation and dislocation slip mechanisms in the matrix.

GRX-810 is an ODS alloy formed in the L-PBF AM process using nanoscale oxide particles (such as yttria) coated L-PBF NiCoCr-base metal powders. The typical metal powder size for L-PBF usage is from 10 to 45 microns and the typical diameter of oxide particles is 50 nm or less. Therefore, the diameter ratio between the metal powder to ceramic powder is around 1000 or more. Let’s assume both yttria and NiCoCr-base metal particles are spherical shape and have uniform size. As surface area of particles is inversely proportional to their diameter, although only small quantity of yttria was used in this study, from 0.3% to 1%, yttria nano particles result in a large total surface area and can effectively cover the entire surface of the NiCoCr-base metal powders even with a low yttria content of 0.3%. For high yttria content, such as above 0.5%, many yttria powders are likely not be able to make direct contact with NiCoCr-base-metal powders. This could increase the difficulty of achieving uniform yttria dispersion into the molten tracks during the laser melting process and may contribute to microdefects and pore formation in the L-PBF NHT parts. In addition, melt pool dynamics during L-PBF rapid solidification and the wetting behavior between yttria particles and the NiCoCr molten matrix may strongly influence particle redistribution, oxide dispersion stability, and local defect formation. These AM-specific phenomena are expected to play an important role in determining the final microstructure and mechanical response of GRX-810, but their direct evaluation requires dedicated high-resolution characterization and is beyond the scope of the present study. This surface-area-based interpretation provides a qualitative explanation for why lower yttria contents may achieve more effective particle dispersion, while excessive yttria additions may increase the likelihood of particle agglomeration and defect formation during L-PBF processing. However, direct quantitative evidence of particle distribution uniformity, agglomeration thresholds, and interparticle spacing is beyond the scope of the present study and requires dedicated high-resolution microstructural characterization. A more detailed investigation of these mechanisms will be presented in a follow-up study.

## Conclusion

This work elucidates the coupled roles of yttria addition and HIP treatment in governing the thermal and mechanical behavior of L-PBF-fabricated NiCoCr based GRX-810 alloys. Across eight compositions (0.3–1 wt% Y_2_O_3_), thermal diffusivity exhibits negligible sensitivity to yttria content, with all measured variations remaining within the ~ 3% instrumental uncertainty of thermal analysis, indicating that heat diffusion is largely insensitive to yttria concentration in this regime.

Thermal conductivity, however, shows a measurable dependence on both composition and post-processing. Quantitative analysis of the thermal conductivity reveals that the standard deviation among different yttria concentrations is ~ 0.6–1.4 W m^−1^ K^−1^, corresponding to ~ 4.5–5.3% for NHT samples and ~ 1.8–3.2% for HIP-processed samples over most of the investigated temperature range. The larger scatter in NHT samples exceeds the nominal instrumental uncertainty and is attributed not only to uncertainty propagation from specific heat measurements, but also to microstructural heterogeneity including porosity, grain boundary density, and dispersoid distribution, which strongly influence electron and phonon scattering during thermal transport. HIP significantly suppresses this variability by reducing porosity and improving microstructural homogeneity.

Despite this measurement-related dispersion, a consistent compositional trend is resolved: increasing yttria content leads to a modest but systematic reduction in thermal conductivity, while a non-monotonic dependence is also present, with the alloy containing ~ 0.35 wt% yttria exhibiting the highest conductivity. Thermal conductivity increases monotonically with temperature for all compositions, and HIP further enhances heat transport at elevated temperatures. At 900 °C, HIP treatment results in an average increase in thermal conductivity of ~ 1.45 W m^−1^ K^−1^ (~ 5.5%) compared with NHT samples.

Mechanical testing reveals that yttria content exerts a strong and non-linear influence on ductility, whereas yield strength is markedly reduced by HIP but remains largely independent of yttria concentration. HIP reduces the yield strength from ~ 785 MPa in the NHT state to ~ 685 MPa, mainly due to grain coarsening, recrystallization, reduced dislocation density, and residual stress relaxation. At the same time, pore closure, microcrack healing, and improved microstructural homogeneity significantly enhance tensile ductility. The ultimate tensile strength of NHT GRX-810 samples with an yttria content less than 0.45% is around ~ 880 MPa and the ultimate tensile strength of HIP samples with an yttria content higher than 0.5% is around ~ 825 MPa. HIP samples with yttria content less than 0.45% exhibit a high ductility at approximately 40% maximum elongation, while HIP samples with yttria content higher than 0.5% exhibit a moderate ductility of approximately 18% maximum elongation at failure.

These results reveal a coupled structure–property relationship in GRX-810 alloys. Yttria content affects particle dispersion during L-PBF processing, which influences oxide agglomeration, defect formation, and local porosity evolution. These microstructural features govern both thermal transport and tensile behavior by altering electron/phonon scattering, crack sensitivity, and deformation compatibility. HIP further modifies this relationship by reducing defect sensitivity and improving microstructural stability; possible contributions from pore closure, microcrack healing, grain coarsening, recrystallization, and residual stress relaxation are consistent with prior studies on HIP-treated L-PBF Ni-based alloys, but their quantitative verification will be the focus of an ongoing follow-up microstructural study.

Together, these results demonstrate that yttria concentration has limited impact on thermal diffusivity but plays a decisive role in tuning thermal conductivity and ductility, while HIP serves as a critical post-processing step to stabilize thermal transport and significantly improve mechanical reliability. This integrated understanding provides quantitative guidance for optimizing GRX-810 alloys through composition–processing co-design for high-temperature, high-performance applications. In the present dataset, the low-yttria regime (0.30–0.45 wt% Y_2_O_3_) provides the most favorable strength–ductility balance. Future work should further investigate the effects of melt pool dynamics, particle wetting behavior, and oxide redistribution during rapid solidification to better establish the AM-specific mechanisms governing oxide dispersion stability and defect formation in GRX-810 alloys.

## Data Availability

The datasets generated and/or analyzed during the current study are available from the corresponding author on reasonable request.
